# Herbal Decoctions for the Levels of Sulfur Dioxide, Benzopyrene, and Mycotoxin from Traditional Korean Medicine Clinics: A Preliminary Study

**DOI:** 10.3390/ijerph192013595

**Published:** 2022-10-20

**Authors:** Hye In Jeong, Ji-Eun Han, Byung-Cheul Shin, Soobin Jang, Jae-Hee Won, Kyeong Han Kim, Soo-Hyun Sung

**Affiliations:** 1 Department of Preventive Medicine, College of Korean Medicine, Kyung Hee University, Seoul 02447, Korea; 2 Department of Policy Development, National Institute of Korean Medicine Development, Seoul 04554, Korea; 3 Division of Clinical Medicine, School of Korean Medicine, Pusan National University, Yangsan 50612, Korea; 4 Department of Preventive Medicine, College of Korean Medicine, Daegu Haany University, Gyeongsan 38609, Korea; 5 Department of Public Infrastructure Operation, National Institute of Korean Medicine Development, Seoul 04554, Korea; 6 Department of Preventive Medicine, College of Korean Medicine, Woosuk University, Jeonju 54986, Korea

**Keywords:** herbal decoction, quality control, safety management standard, sulfur dioxide, benzopyrene, mycotoxin

## Abstract

In this study, we investigated whether the levels of sulfur dioxide (SO_2_), benzopyrene, and mycotoxins in herbal decoctions in Korea in 2019 were within normal limits. In total, 30 decoctions composed of multi-ingredient traditional herbs were sampled from traditional Korean medicine (TKM) clinics, TKM hospitals, and external herbal dispensaries in 2019. The decoctions were analyzed for SO_2_, benzopyrene, and mycotoxins using 10 samples. SO_2_ and benzopyrene were not detected in any of the herbal decoctions. With regard to mycotoxins, aflatoxin B1 was not detected, but B2 was detected in 7 cases (0.00~0.04 ppb), G1 in 13 cases (0.03~0.29 ppb), and G2 in 9 cases (0.02~0.93 ppb). None of these values exceeded the restrictions in prior studies. Thus, we confirm that the amounts of SO_2_, benzopyrene, and mycotoxins in herbal decoctions are at safe levels and provides the basis of establishing safety management criteria for herbal decoctions.

## 1. Introduction

Herbal medicine is a widely utilized treatment that is growing with the global complementary and alternative medicine market [1]. Herbal medicines are used to prevent diseases and promote personal health. Complaints about existing treatments, positive aspects related to herbal medicine, and traditions of family are reasons why people prefer herbal medicine [2].

However, there have been reports about heavy metals, aflatoxins, benzopyrene, and microorganisms in herbal medicines including decoctions around the world [3,4,5,6]. A systematic review by Posadzki et al. [7] found that herbal medicinal products were contaminated or adulterated with insects, rodents, parasites, dust, pollen, microbes, fungi, pesticides, toxins, heavy metals, and so on. Severe side effects such as hepatic encephalopathy, meningitis, multi-organ failure, and death were caused by these contaminants. Although inhalation is the major way of exposing humans to sulfur dioxide (SO_2_), humans can be exposed through ingestion. SO_2_ is a severe irritant to the eyes, mucous membranes, epidermis, and other organs [8]. Benzopyrene may cause an acute burning or rash feeling in body parts. Prolonged exposure to benzopyrene can cause skin or lung cancer in humans and damage the developing fetus [9]. Mycotoxins can affect humans in various ways, including neurologic impairment and failure of the liver, kidney, or heart failure. Aflatoxins, known as carcinogens, are potent toxins and also lead to liver impairment in animals [10].

Although herbal medicine has an effect of treating several illnesses, many countries use it continuously at uncontrolled levels [11]. Herbal medicine is a constantly increasing industry and its popularity is international, so more effort should be put into policy to minimize the safety risks and provide scientific evidence [12].

According to the World Health Organization (WHO), in 2018, about 90% of member states and countries had regulations about herbal medicines [13]. Many countries have suggested guidelines on good manufacturing practices (GMP) and methods to standardize the quality of herbal medicines and to make them safe and efficient [14]. However, most guidelines of herbal medicine often follow rules for pharmaceutical products, foods, or food supplements without special consideration [12]. In addition, most countries have no quality control system for herbal decoctions.

Herbal decoctions require strict control of contamination and manufacturing processes [11]. Safety concerns from contamination, heavy metals, and adulteration arise mainly from restricted quality control regulations and deficiency of internationally accepted standards [12]. Reproducible results demand the development, evaluation, and standardization of the latest methods of chemistry, biology, and pharmacology [14].

In the case of Korea, a pilot project to apply herbal decoctions to health insurance has been implemented. However, there is no safety management standard to verify the quality of herbal decoctions. Therefore, the government has attempted to provide guidelines for herbal decoctions. We monitored the quality control of the herbal decoctions as part of this study. We examined and reported on heavy metal and pesticide residue contents in our previous study [15]. In this study, we aimed to investigate herbal decoctions in Korea for SO_2_, benzopyrene, and mycotoxin to suggest evidence for establishing safety criteria at the government level.

## 2. Materials and Methods

### 2.1. Sample Collection

The ten types of decoctions, including Galgeuntang which is commonly used in TKM institutions [16], based on the traditional Korean medicine (TKM) Consumption Statistics Survey 2017, were tested to examine them for SO_2_, benzopyrene, and mycotoxin content. The formulae and compositions of them have been provided in a previous study [15]. For this study, thirty herbal decoction pouches prescribed by TKM clinics, TKM hospitals, and external herbal dispensaries were obtained from October to December 2019 and were stored in a refrigerator. A total of 10 decoction pouches for each institute were collected when the volume was 80 mL and were not collected when the volume was less than 80 mL.

### 2.2. Standards and Reagents

The reagents used for the SO_2_ test were methyl red, 30% hydrogen peroxide solution, hydrochloric acid, 0.01 N sodium hydroxide solution (Factor 1.00, Wako, Tokyo, Japan), ethanol (High-performance liquid chromatography (HPLC) grade, J. T. Baker, Phillipsburg, NJ, USA), and nitrogen gas (purity 99.995%). SO_2_ was extracted as the test apparatus using a Monier-Williams transformation apparatus and an automatic potentiometer (T50M, Mettler-Toledo, Zurich, Switzerland) was used.

Aflatoxin B1, B2, G1, and G2 mixed solutions (Aflatoxin Mix, Sigma, Burlington, MA, USA) were used as standards for the mycotoxin test. Pididium hydrobromic acid perbromate (PBPB, Sigma, USA) was used as the derivatization reagent; methanol (HPLC grade, J. T. Baker, USA), acetonitrile (HPLC grade, J. T. Baker, USA), acetic acid (HPLC grade, J. T. Baker, USA), and Tween 20 were used as solvents. (Bio-Rad, Hercules, CA, USA); and an immunoaffinity column (AflaTest, Vicam, Manchester, TN, USA) was used.

The standard used for the benzopyrene test was benzopyrene (Sigma, USA). Furthermore, 3-methylcholanthrene (Sigma, USA) was used as an internal standard, and n-hexane (HPLC grade, J. T. Baker, USA), acetonitrile (HPLC grade, J. T. Baker, USA), anhydrous sodium sulfate (HPLC grade, J. T. Baker, USA), dichloromethane (J. T. Baker, USA), and a Florisil cartridge (1 g, Sep-Pak^®^ Waters, Bedford, MA, USA) were used.

### 2.3. Experimenting Methods

#### 2.3.1. Pre-Treatment of Samples

For the testing SO_2_, benzopyrene, and mycotoxins, all testing solutions were prepared in accordance with the Korea Pharmacopoeia (KP) General Testing Process [17].

#### 2.3.2. Analysis Instrument and Analysis Conditions

**Figure 1:**
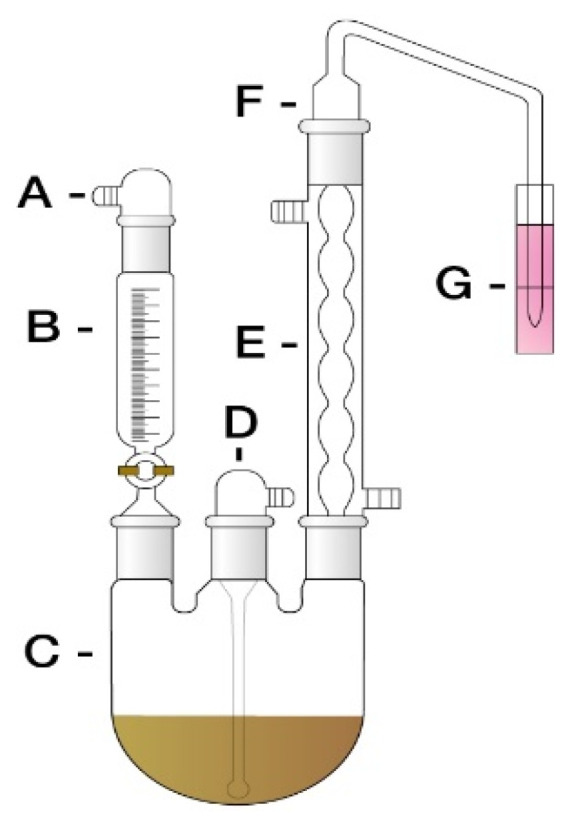
Sulfur dioxide (SO_2_) extraction device. A: Inlet Adapter, B: Addition Funnel, C: 3-N Flask, D: Gas Inlet Adapter, E: Allihn Condenser, F: Bubbler Tube, G: Cylinder.

The SO_2_ test adhered to the Korean Pharmacopoeia [17]. Purity Test of Herbal Medicine Test Method SO_2_ test method [17]. The amount of SO_2_ was calculated using an automatic potentiometer for the collected solution using an SO_2_ extraction device (Figure 1) according to the Monier-Williams method [18,19]. In the Monier-Williams method [18,19], SO_2_ is released from sulphites and some bound compounds when a sample is mixed with an acid normally hydrochloric acid, but sometimes phosphoric acid and heated. The SO_2_ is distilled using a stream of nitrogen gas which carries the gaseous SO_2_ into an absorbing solution of hydrogen peroxide (H_2_O_2_) where it is oxidised to sulphuric acid. The amount of SO_2_ distilled into the H_2_O_2_ is determined by titration with 0.1 M sodium hydroxide. Moreover, 1 mL of 0.1 M NaOH is equivalent to 3.203 mg SO_2_.

HPLC was used to analyse mycotoxins and benzopyrene [17,20,21]. The instrument analysis conditions are listed in Table 1 and Table 2.

### 2.4. Validation of the Test Method

The recovery rate was calculated by following method: A sample with no detectable analytical material was selected and then the standard solution was added. This process was repeated three times. The difference between the concentrations of the samples including the standard and the control was calculated. The limit of detection (LOD) and the limit of quantitation (LOQ) were calculated using the following formulas, consisting of the standard deviation of the reaction (σ) and the gradients of the measurement graph (S). The average of three repetitions of the measured standard solutions were applied in a stepwise manner.

LOD = 3.3 × σ/S, LOQ = 10 × σ/S

σ: the mean standard deviation; S: the individual slop.

#### 2.4.1. Recovery Rate

The recovery rates of the SO_2_ tests were measured for each concentration using potassium metabisulfite (Table 3). The recovery rate test of the mycotoxin was performed such that the final detection concentrations were 5, 10, and 20 ppb (Table 4). The recovery rate of the benzopyrene test was measured such that the final detection concentration was 20 ppb of benzopyrene (Table 5).

#### 2.4.2. Detection Limit (LOD), and Quantitation Limit (LOQ) of the Analytical Equipment

The LOD and LOQ of analysis equipment are listed in Table 6 and Table 7.

### 2.5. Test Criteria

The test items and safety management criteria for SO_2_, mycotoxin, and benzopyrene were developed in accordance with the findings of literature reviews and consensus among experts. More details on the development process can be found in a previous paper [22]. SO_2_ should be ≤30 ppm, the mycotoxin standard should be ≤15 ppb of total aflatoxin, and ≤10 ppb of aflatoxin B1. The concentration of benzopyrene should be ≤5 ppb.

## 3. Results

### 3.1. Sulfur Dioxide (SO_2_)

SO_2_ content was analyzed in 10 samples of decoctions frequently prescribed in Korea collected from TKM clinics, TKM hospitals, and external herbal dispensaries, respectively, making a total of 30 samples. No SO_2_ content was detected in any of the samples (Table 8).

### 3.2. Mycotoxin

Mycotoxin detection was analyzed in 10 samples of decoctions frequently prescribed in Korea collected from TKM clinics, TKM hospitals, and external herbal dispensaries, respectively, making a total of 30 samples. Aflatoxin B1 was not detected in any samples, B2 was detected in 7 cases (0.00~0.04 ppb), G1 in 13 cases (0.03~0.29 ppb), and G2 in 9 cases (0.02~0.93 ppb). The total aflatoxin content of all herbal decoctions was ≤0.96 ppb (Table 9).

### 3.3. Benzopyrene

Benzopyrene was analyzed in 10 samples of decoctions frequently prescribed in Korea collected from TKM clinics, TKM hospitals, and external herbal dispensaries, respectively, making a total of 30 samples. Benzopyrene was not detected in any of the herbal decoctions (Table 10).

## 4. Discussion

According to the previous study [22], a set of safety management standards on SO_2_, benzopyrene, and mycotoxins was identified through the literature reviews (e.g., the WHO guidelines for regulation of herbal medicines, Japanese pharmacopeia, Japanese standards for nonpharmacopeial crude drugs, pharmacopeia of People’s Republic of China, the US botanical drugs guidance, European pharmacopeia, and a quality control guideline for herbal or botanical medicine extracts of Korea) and the expert consensus.

The amount of SO_2_ was found to be less than the quantitative or detection limit in all herbal decoctions. It appears that SO_2_ would have been washed away during washing processes, even if it was previously present in the decoctions [23]. In Yu’s study, SO_2_ was detected in only 1 (0.7%) out of 155 samples of herbal decoctions, and the level of sulfites was high (17.6 mg/kg) in the same sample for unknown reasons [24].

With regard to mycotoxins, aflatoxin was detected in 18 samples. The concentrations of aflatoxin in 17 samples were below 0.3 ppb, which is low. However, in SSanghwatang from a certain Korean medicine hospital, 0.96 ppb of aflatoxin was detected. Aflatoxins are secondary metabolites produced by different fungal strains such as *Aspergillus flavus* and *Aspergillus parasiticus*. Aflatoxin B1 is the most recurrent and harmful toxin. Aflatoxin B1 manifests a wide range of cytotoxicity in neuronal cells [25] and is considered to be carcinogenic (Group 1) and potentially carcinogenic for humans (Group 2B) [26].

According to a study from Pakistan on aflatoxin content in herbal formulations, total Aflatoxins were detected in 52.5% of analysed samples, with Aflatoxin B1 being the most frequently occurring (46.3%), followed by Aflatoxin G1 (35.6%), Aflatoxin B2 (34.5%), and Aflatoxin G2 (27%) [6]. In a study conducted in Nigeria, aflatoxin contamination was discovered in 48 (84.21%) out of 57 herbal medicine samples [27]. B1 was not detected in this study, and the results for G1, B2, and G2 were similar. Aflatoxin was managed by applying criteria only to 20 kinds of raw herbs as of 2022. Even though Aflatoxin B1 was not detected in these analyses, additional standards for the management of herbal decoctions would be necessary to eliminate the risk of neurotoxicity.

Benzopyrene was not detected in any sample. Polycyclic aromatic hydrocarbons can be generated from the incomplete combustion of organic matter or produced when organic sediments are chemically transformed into fossil fuels such as oil and coal [28]. Benzo[a]pyrene is considered to be a Group 1 compound (carcinogenic to humans). In December 2009, the Korea Food and Drug Administration established a benzopyrene standard of less than 5 μg/kg for all herbal medicines (except for mineral herbal medicines), and they also announced a test method [29].

Because of the potential health risks, the WHO and some countries such as the United States and Europe developed a program to manage the quality of herbal medicine [30,31,32]. Although there are many regulations on herbal medicines in Korea, such as GMP, the herbal decoction lacks a safety management standard. The herbal decoction generally undergoes a packing process after extraction, which may cause contamination. Hence, it is necessary to check the hygiene management of manufacturing environments and develop guidelines for post-extraction processes. As a member of the Pharmaceutical Inspection Cooperation Scheme (PIC/S), South Korea is required to follow the GMP, good laboratory practice (GLP), and good clinical practice (GCP) guidelines [33,34]. In the manufacture of herbal medicinal products, the PIC/S guide was more detailed than hGMP. Only the PIC/S guide had an exact clause about proper solvent and herbal preparation. According to the PIC/S, regulation should be determined for the relevant guidance on quality, so the regulation in Korea should follow the international guidance quickly.

### Limitation of This Study

This study has several limitations. First, the sample size was small. Since herbal decoctions had to be collected and manufactured on the same day, only 30 samples were analysed in this study. In addition, the regional distribution of samples was relatively uneven. Second, the types of herbal decoctions constituting the 10 prescriptions used in the analysis were all different. Therefore, it was difficult to identify some of the detected factors for SO_2_ and mycotoxin (e.g., preparation environment, composition of herbal medicines, or storage conditions). Third, in some cases, the labels for a single dose were not clear. In the case of herbal medicinal products considered to be pharmaceuticals, there are standardized bottles or packages. It is also necessary to consider applying similar standards to herbal medicinal products and to calculate the total amount of decoctions compared to the amounts of raw herbs. There are still potential risk factors, such as Staphylococcus aureus which can cause shock, so hygiene management is required when preparing decoctions.

## 5. Conclusions

In conclusion, this study examined the content of SO_2_, benzopyrene, and mycotoxins in herbal decoctions from Korean medical institutions. Because these decoctions were made from pharmaceutical-grade herbs, there were no hazardous safety issues. However, systematic complements are needed to meet consumers’ high demand for the safety of herbal decoctions.

## Figures and Tables

**Table 1:** HPLC analysis conditions for mycotoxin.

Instrument	Analysis Condition (Waters, USA 2475 FLD, Alliance 2695)
Column	Xbridge C18 (4.6 × 250 mm, 5 μm)
Mobile phase	Water·Methanol·Acetonitrile (60:25:15)
Flow rate	1.0 mL/min
Injection volume	10 μL
Post-column derivatization system	Post Column Reaction Module (PBPB)
Detector	Fluorescence (Ex: 365 nm Em: 435 nm)

HPLC: high performance liquid chromatography.

**Table 2:** HPLC analysis conditions for benzopyrene.

Instrument	Analysis Condition (Waters, USA 2475 FLD, Alliance 2695)
Column	PAH C18 (4.6 × 250 mm, 5 μm)
Mobile phase	Acetonitrile·Water (8:2)
Flow rate	1.0 mL/min
Injection volume	10 μL
Detector	Fluorescence (Ex: 294 nm Em: 404 nm)

HPLC: high performance liquid chromatography.

**Table 3:** Recovery rate of analysis equipment for SO_2_.

	7.5 ppm	15 ppm	30 ppm	60 ppm
Recovery rate (%)	84.3 ± 5.0	88.3 ± 5.0	88.7 ± 3.1	90.3 ± 5.5

**Table 4:** Recovery rate of analysis equipment for mycotoxin.

		5 ppb	10 ppb	20 ppb
Recovery rate (%)	Aflatoxin B1	88.3 ± 6.2	91.7 ± 6.9	96.8 ± 5.5
	Aflatoxin B2	84.6 ± 1.2	89.3 ± 5.2	96.2 ± 6.8
	Aflatoxin G1	79.7 ± 3.7	89.6 ± 9.4	89.8 ± 5.0
	Aflatoxin G2	78.6 ± 3.2	82.7 ± 3.0	82.8 ± 2.3

**Table 5:** Recovery rate of analysis equipment for benzopyrene.

	Benzopyrene	3-Methylcholanthrene
Recovery rate (%)	105.3 ± 7.4	84.2 ± 6.3

**Table 6:** Detection limit and quantitation limit of analysis equipment for mycotoxin.

	Limit of Detection (ppm)	Limit of Quantitation (ppm)
Aflatoxin B1	0.07	0.24
Aflatoxin B2	0.05	0.18
Aflatoxin G1	0.07	0.22
Aflatoxin G2	0.05	0.15

**Table 7:** Detection limit and quantitation limit of analysis equipment for benzopyrene.

	Limit of Detection (ppm)	Limit of Quantitation (ppm)
Benzopyrene	0.025	0.077

**Table 8:** SO_2_ detection result of herbal decoctions.

Herbal Decoctions	TKM Clinics (ppm)	TKM Hospitals (ppm)	External Herbal Dispensaries (ppm)
Galgeun-tang	0.000	0.000	0.000
Kangwhalyupung-tang	0.000	0.000	0.000
Dangguisu-san	0.000	0.000	0.000
Dokhwalgisaeng-tang	ND	ND	ND
Banhasasim-tang	ND	ND	0.000
Bangpungtongseong-san	ND	ND	ND
Bojungikgi-tang	ND	ND	ND
Sipjeondaebo-tang	ND	ND	ND
Ssanghwa-tang	ND	ND	ND
Ojeok-san	ND	ND	ND
Test criteria	≤30 ppm	≤30 ppm	≤30 ppm

0.000: less than the quantitative limit, ND: less than the detection limit, TKM: traditional Korean medicine, External herbal dispensaries: type of pharmacy that provides various types of herbal medicines to other TKM clinics or hospitals.

**Table 9:** Mycotoxin detection result of herbal decoctions.

Herbal Decoctions		Aflatoxin B1 (ppb)	Aflatoxin B2 (ppb)	Aflatoxin G1 (ppb)	Aflatoxin G2 (ppb)	Total Aflatoxin (ppb)
TKM Clinics	Galgeun-tang	ND	ND	ND	0.05	0.05
	Kangwhalyupung-tang	ND	ND	0.04	0.02	0.05
	Dangguisu-san	ND	0.01	0.12	0.02	0.15
	Dokhwalgisaeng-tang	ND	0.00	0.05	0.03	0.08
	Banhasasim-tang	ND	ND	ND	ND	ND
	Bangpungtongseong-san	ND	ND	ND	0.03	0.03
	Bojungikgi-tang	ND	ND	0.29	ND	0.29
	Sipjeondaebo-tang	ND	0.01	0.05	ND	0.06
	Ssanghwa-tang	ND	ND	0.03	0.93	0.96
	Ojeok-san	ND	ND	ND	ND	ND
TKM Hospitals	Galgeun-tang	ND	ND	ND	ND	ND
	Kangwhalyupung-tang	ND	ND	ND	ND	ND
	Dangguisu-san	ND	ND	ND	ND	ND
	Dokhwalgisaeng-tang	ND	0.04	0.05	ND	0.09
	Banhasasim-tang	ND	ND	ND	ND	ND
	Bangpungtongseong-san	ND	ND	ND	ND	ND
	Bojungikgi-tang	ND	ND	ND	ND	ND
	Sipjeondaebo-tang	ND	0.02	0.11	ND	0.12
	Ssanghwa-tang	ND	ND	0.05	ND	0.05
	Ojeok-san	ND	ND	0.06	ND	0.07
External Herbal Dispensaries	Galgeun-tang	ND	ND	ND	ND	ND
	Kangwhalyupung-tang	ND	ND	ND	ND	ND
	Dangguisu-san	ND	ND	ND	ND	ND
	Dokhwalgisaeng-tang	ND	ND	ND	ND	ND
	Banhasasim-tang	ND	ND	ND	ND	ND
	Bangpungtongseong-san	ND	ND	ND	ND	ND
	Bojungikgi-tang	ND	ND	ND	ND	ND
	Sipjeondaebo-tang	ND	ND	ND	ND	ND
	Ssanghwa-tang	ND	ND	ND	ND	ND
	Ojeok-san	ND	ND	ND	ND	ND
Test criteria		≤15 ppb of total aflatoxin and ≤10 ppb of aflatoxin B1				

ND: less than the detection limit, TKM: traditional Korean medicine, External herbal dispensaries: type of pharmacy that provides various types of herbal medicines to other TKM clinics or hospitals.

**Table 10:** Benzopyrene detection result of herbal decoctions.

Herbal Decoctions	TKM Clinics (ppb)	TKM Hospitals (ppb)	External Herbal Dispensaries (ppb)
Galgeun-tang	ND	ND	ND
Kangwhalyupung-tang	ND	ND	ND
Dangguisu-san	ND	ND	ND
Dokhwalgisaeng-tang	ND	ND	ND
Banhasasim-tang	ND	ND	ND
Bangpungtongseong-san	ND	ND	ND
Bojungikgi-tang	ND	ND	ND
Sipjeondaebo-tang	ND	ND	ND
Ssanghwa-tang	ND	ND	ND
Ojeok-san	ND	ND	ND
Test criteria	≤5 ppb	≤5 ppb	≤5 ppb

ND: less than the detection limit, TKM: traditional Korean medicine, External herbal dispensaries: type of pharmacy that provides various types of herbal medicines to other TKM clinics or hospitals.

## Data Availability

The data will be made available upon reasonable request.

## Abbreviations

GCP; good clinical practice, GMP; good manufacturing practices, GLP; good laboratory practice, HPLC; high-performance liquid chromatography, KP; Korea Pharmacopoeia, LOD; limit of detection, LOQ; limit of quantitation, PIC/S; Pharmaceutical Inspection Cooperation Scheme, SO_2_; sulfur dioxide, TKM; traditional Korean medicine, WHO; World Health Organization.
